# Phonon transport assisted by inter-tube carbon displacements in carbon nanotube mats

**DOI:** 10.1038/srep02774

**Published:** 2013-09-27

**Authors:** Assel Aitkaliyeva, Di Chen, Lin Shao

**Affiliations:** 1 Materials Science and Engineering Program, Texas A&M University, College Station, Texas, 77843, USA; 2 Department of Nuclear Engineering, Texas A&M University, College Station, Texas, 77843, USA

## Abstract

Thermal transport in carbon nanotube (CNT) mats, consisting of randomly networked multi-walled carbon nanotubes (MWNTs), is not as efficient as in an individual CNT because of the constrained tube-to-tube phonon transport. Through experiments and modeling, we discover that phonon transport in CNT mats is significantly improved by ion irradiation, which introduces inter-tube displacements, acting as stable point contacts between neighboring tubes. Inter-tube displacement-mediated phonon transport enhances conductivity, while inter-tube phonon-defect scattering reduces conductivity. At low ion irradiation fluence, inter-tube thermal transport enhancement leads to thermal conductivity increase by factor > 5, while at high ion irradiation fluence point defects within tubes cause a decrease in thermal conductivity. Molecular dynamics simulations support the experimentally obtained results and the proposed mechanisms. Further conductivity enhancement in irradiated mats was obtained by post-irradiation heat treatment that removes majority of the defects within the tubes without affecting thermally stable inter-tube displacements.

Different from their bulk counterparts, low dimensional carbon systems, including graphene, carbon onions and CNTs, have unique radiation response due to their size and quantum mechanics effects, and their mechanical, thermal and electrical properties can be modified by ion or electron irradiations^1^,^2^. Irradiation creates non-equilibrium defects, including Stone-Wale defects, carbon adatoms, vacancies, and vacancy complexes, in low dimensional carbon materials^3^. Defect migration, clustering and local rearrangements can result in unusual structural changes. For example, electron irradiation can perforate the outer shell of tubes, bend the tubes and adjust their diameters^4^. Transformation of multi-walled nanotubes (MWNTs) into highly ordered pillbox-like nanocompartments^5^, and transformation of a single-walled nanotube (SWNT) bundle into a MWNT have been observed in previous irradiation studies^4^. Irradiation can induce high pressure inside carbon onions and carbon nanotubes, thus leading to phase changes of materials inside the cell and tube cores^6^,^7^. Ion and electron beams can be used to thin, slice, cut CNTs^8^, or weld two adjoining CNTs^9^. In CNT films/mats, electron or ion irradiation can induce inter-tube linking by introducing one fourfold coordinated interstitial atom between two touching tubes^10^. Both experimental and modeling studies have shown that inter-tube linking can significantly improve mechanical properties of CNT bundles/mats^10^,^11^.

CNTs have superior thermal conductivity, which makes them ideal for heat management in microelectronics. Modeling suggested that the thermal conductivity of an isolated SWNT can reach 6.6 × 10^6^ W/mK^12^. A small amount of defects in CNTs, however, could lower thermal conductivity by orders of magnitude^13^. It has been shown that mono-and di-vacancies and Stone-Wales defects are most effective in scattering intermediate and high frequency phonons^13^. Phonon transport in irradiated CNTs, even when they are several micrometer long, is more ballistic-like^14^. Phonon scattering from defects is believed to be one major causes of large variation in experimentally measured thermal conductivities, which range from 300 to 7000 W/mK^12^,^13^,^14^,^15^,^16^. Thermal properties of CNT mats/films/buckypapers are lower than that of individual CNTs because of the high thermal resistance between tubes. Along the axis of CNTs, thermal conductivity of aligned CNT films at room temperature was measured to be 35 to 250 W/mK^17^,^18^, while off-axis thermal conductivity of CNTs mats was typically one order of magnitude lower^19^. For randomly networked CNT mats, thermal conductivities as low as 0.2 W/mK were reported^20^.

The present study is motivated to improve thermal properties of randomly networked CNT mats. Through an integrated experimental and modeling study, we show that C atoms, displaced and trapped between adjacent tubes, become stable point contacts linking tubes and play an important role in phonon transport. Hydrogen (H) ion beam of three energies (1.5, 2, and 3 MeV) was used to irradiate CNT mats to fluences ranging from 2 × 10^14^ to 6 × 10^15^/cm^2^. Projected ranges of H ions, calculated using binary collision approximation Monte Carlo simulation code SRIM^21^, are 107 μm (1.5 MeV), 170 μm (2 MeV), and 335 μm (3 MeV), so H ions will pass through 100 μm thick CNT mats without introducing chemical effects. The highest fluence to which specimens are irradiated in the present study corresponds to 8.4 × 10^−5^ dpa (displacements per atom), which does not exceed the reported amorphization threshold value of 0.15 dpa^22^. Thus, ordered structure is preserved even at the highest ion fluence at any selected ion energy. No mat densification (Supplementary Fig. 1a, b, c, d) and no amorphization were observed in this study (Supplementary Fig. 2a, b, c, d).

## Results

**Figure 1:**
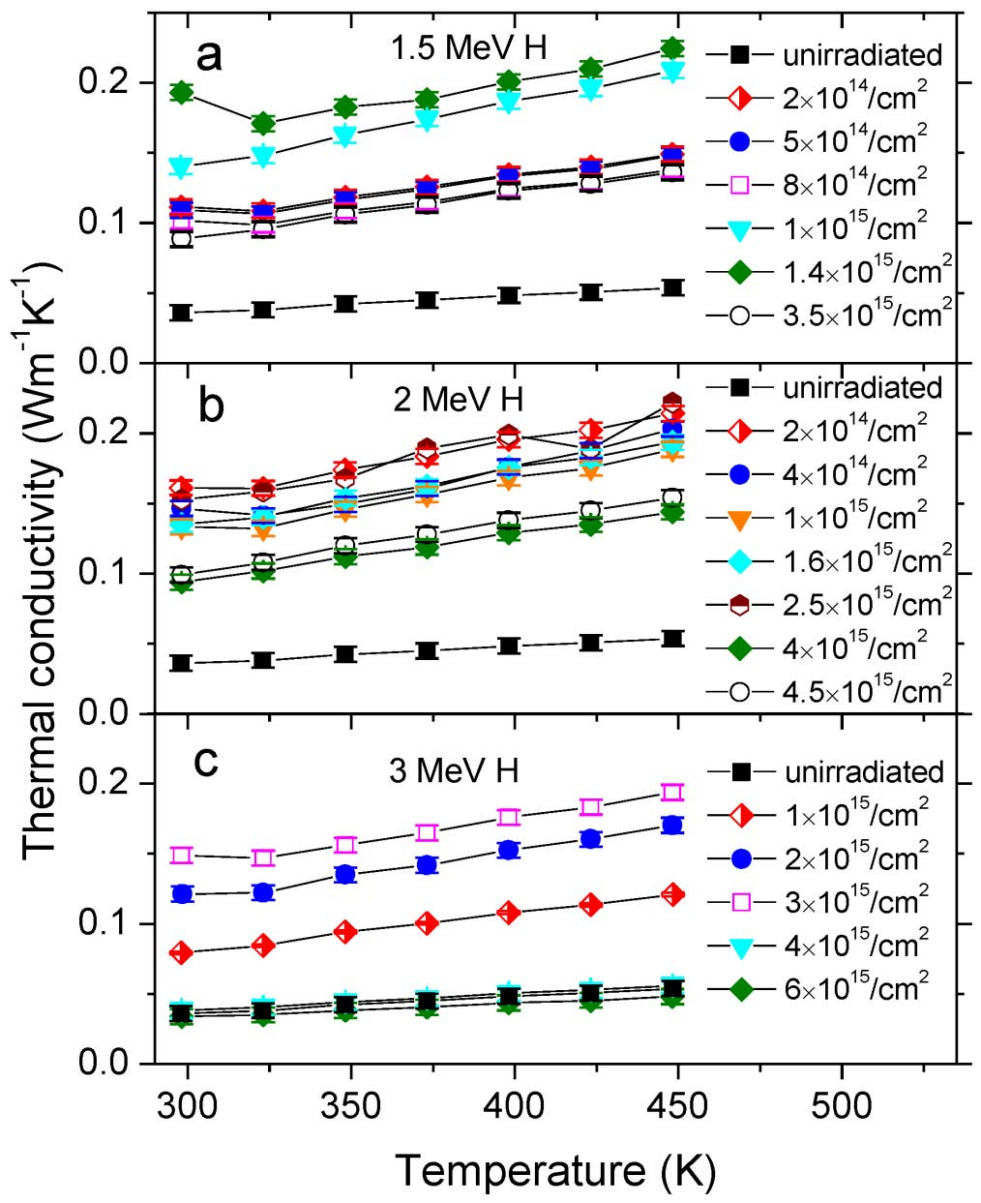
Thermal conductivities of CNT mats as a function of temperature. Thermal conductivities after irradiation with (a), 1.5 MeV H ions to fluences ranging from 2 × 10^14^/cm^2^ to 3.5 × 10^15^/cm^2^. (b), 2 MeV H ions to fluences ranging from 2 × 10^14^/cm^2^ to 4.5 × 10^15^/cm^2^. (c), 3 MeV H ions to fluences ranging from 1 × 10^15^/cm^2^ to 6 × 10^15^/cm^2^. Thermal conductivities of unirradiated CNT mat are provided as a reference. The error bars were extracted from 5 repeated measurements.

Figure 1 shows the temperature dependence of thermal conductivities (κ) of CNT mats before and after irradiation. The κ values were extracted from experimentally measured thermal diffusivities (Supplementary Fig. 3a, b, c). Prior to irradiation conductivity value was at about 0.04 W/mK at 300 K. Irradiation with 1.5 MeV H ions to 1.4 × 10^15^/cm^2^ increases κ at 300 K to 0.19 W/mK. However, further irradiation to 3.5 × 10^15^/cm^2^ resulted in reversal of the trend. As shown in Fig. 1b and 1c, analogous conductivity enhancement at low fluences, followed by reduction at high fluences was observed in CNT mats irradiated with 2 MeV and 3 MeV H ions. This agreement among all irradiation conditions affirms the existence of ion fluence range in which conductivity can be enhanced most. Thermal conductivity of specimens exhibits weak temperature dependence: slight increase with temperature can be seen. This suggests that in CNT mats, both irradiated and unirradiated, transition from phonon-defect scattering (increase of κ with increasing temperature) to phonon-phonon scattering (decrease of κ with temperature) occurs at temperatures exceeding 450 K.

Previous experimental studies reported κ of ~ 0.2 W/mK^20^, and modeling predicted κ to be in the range of ~ 0.8 to 1.8 W/mK (for a density of 0.5 g/cm^3^) in unirradiated mats^23^. Since κ values are sensitive to CNT type, lengths, and diameters^23^, the relatively low κ value measured in this study, 0.04 W/mK, could be caused by the difference in CNT starting materials and in thermal-mechanical processing (hot pressing) used in the mat fabrication.

**Figure 2:**
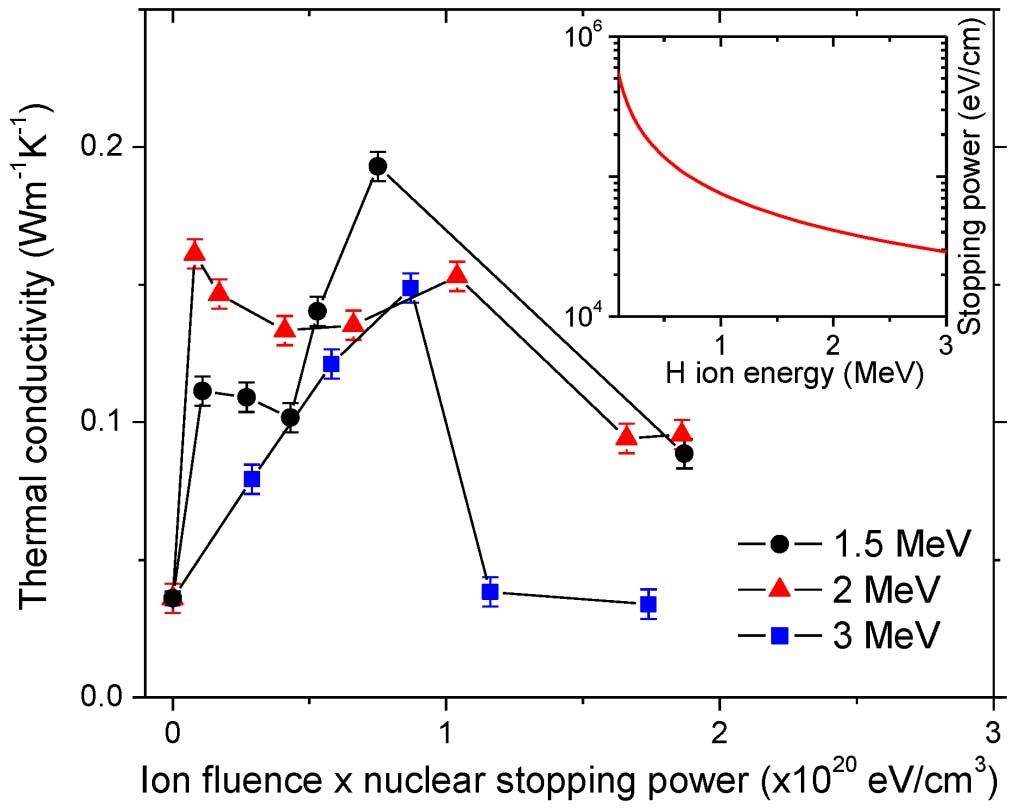
Thermal conductivities of CNT mats as a function of radiation damage levels. The conductivities were measured at 300 K and plotted as a function of ion fluence multiplied by nuclear stopping power, which corresponds to the density of energy deposited in the sample by nuclei-nuclei scattering. The inset shows nuclear stopping power, *dE*/*dx*, vs. *E*, where *E* is energy and *x* is ion penetration depth. The error bars were extracted from 5 repeated measurements.

Due to strong covalent *sp*^*2*^ bonding, heat transfer in CNTs is mainly mediated by phonons, with almost negligible contribution from electrons^15^. Therefore, production of displacements by nuclei-nuclei scattering is responsible for changes in thermal properties; the change is dominated by nuclear stopping of H ions. Nuclear stopping power of H in carbon is energy dependent, with a maximum attained around 200 eV. The nuclear stopping power values of H ions in CNTs are calculated to be 5.3 × 10^4^, 4.1 × 10^4^, and 3.0 × 10^4^ eV/cm at 1.5, 2 and 3 MeV, respectively. Thus fluences required for obtaining maximum κ enhancement shift to higher values as H ion energy is increased from 1.5 to 3 MeV. As shown in Fig. 2, conductivity values at 300 K for all three irradiation energies show consistent displacement dependence when ion fluences are normalized by multiplying to corresponding nuclear stopping power values.

**Figure 3:**
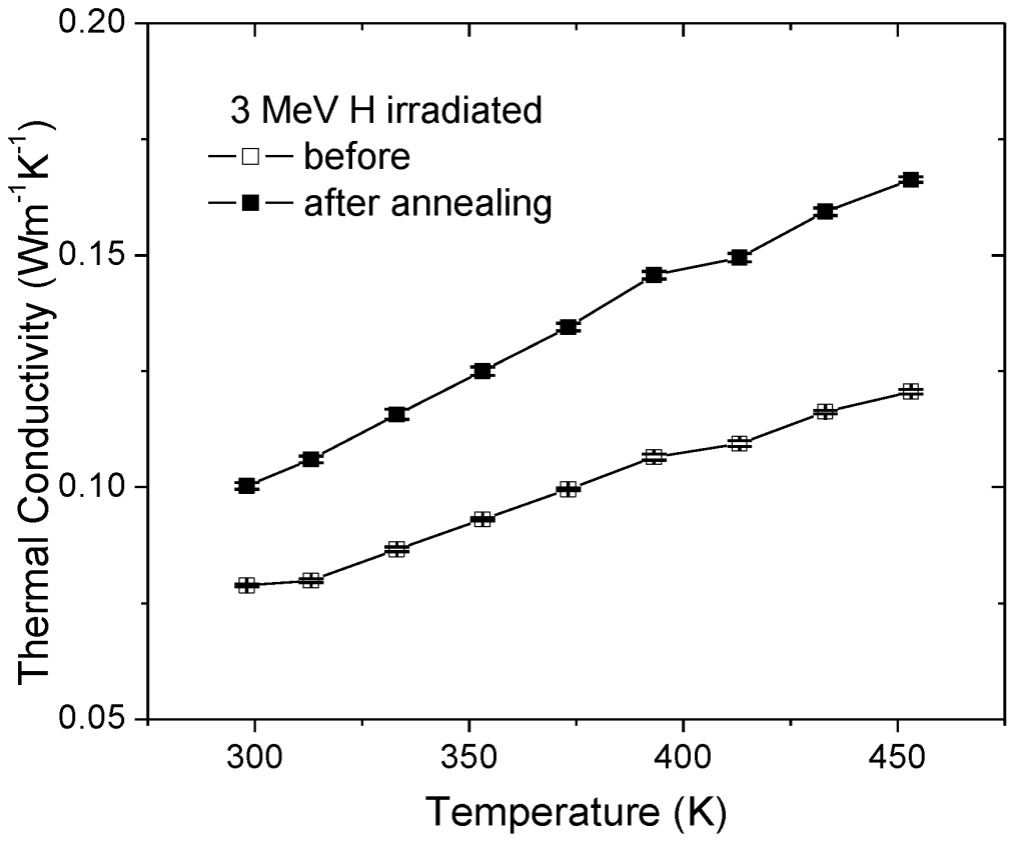
Thermal conductivities of CNT mats before and after post-irradiation annealing at 1173 K for 15 min: Prior to the annealing, mats were irradiated to 2 × 10^15^/cm^2^ with 3 MeV H ions. The error bars were extracted from 5 repeated measurements.

Post-irradiation heat treatment can achieve additional thermal conductivity enhancement. Previous studies suggest defect removal during post-irradiation annealing requires low activation energy of 0.36 eV^24^. Therefore the annealing temperature of 1173 K selected in present study was high enough to cause defect annealing. Figure 3 shows that κ values of CNT mat, irradiated with 3 MeV H ions to a fluence of 2 × 10^15^/cm^2^, increase from 0.08 W/mK to 0.1 W/mK after annealing at 1173 K for 15 minutes. The enhancement can be explained by in-plane defect removal within the tubes, which reduces phonon scattering. Migration energy of C adatoms is estimated to be 0.4 eV^25^ or less^1^, and the high mobility of adatoms leads to defect removal during annealing^25^. In comparison, migration energy of interstitial C atoms trapped between graphene planes is calculated to be 2.12 eV^26^, or > 1.5 eV^27^. This suggests that displaced carbon atoms confined by adjacent tubes, or by neighboring walls within a MWNT, are less mobile and their removal requires higher annealing temperatures. The difference in defect removal efficiencies suggests that upon annealing phonon transport across the tubes remains efficient and defect-phonon scattering within the tube is reduced. Both effects lead to further κ enhancement after post-irradiation annealing, as observed in Fig. 3. The room temperature κ value was 0.12 W/mK for irradiated mats, and it increased to 0.16 W/mK after the annealing. Measured κ values shown in Fig. 3 are systematically lower than that shown in Fig. 1c, even though ion irradiation was conducted to the same fluence of 2 × 10^15^/cm^2^ and at the energy of 3 MeV. This is caused by κ variation among different mats. Conductivity values were found to be sensitive to hot pressing process used in mat fabrication and the mats used to acquire results shown in Fig. 1c and Fig. 3 came from different batches. Such difference should not cause issues in our comparison studies since all mats involved in one figure were originally cut from the same parent mat prior to annealing and/or irradiation.

**Figure 4:**
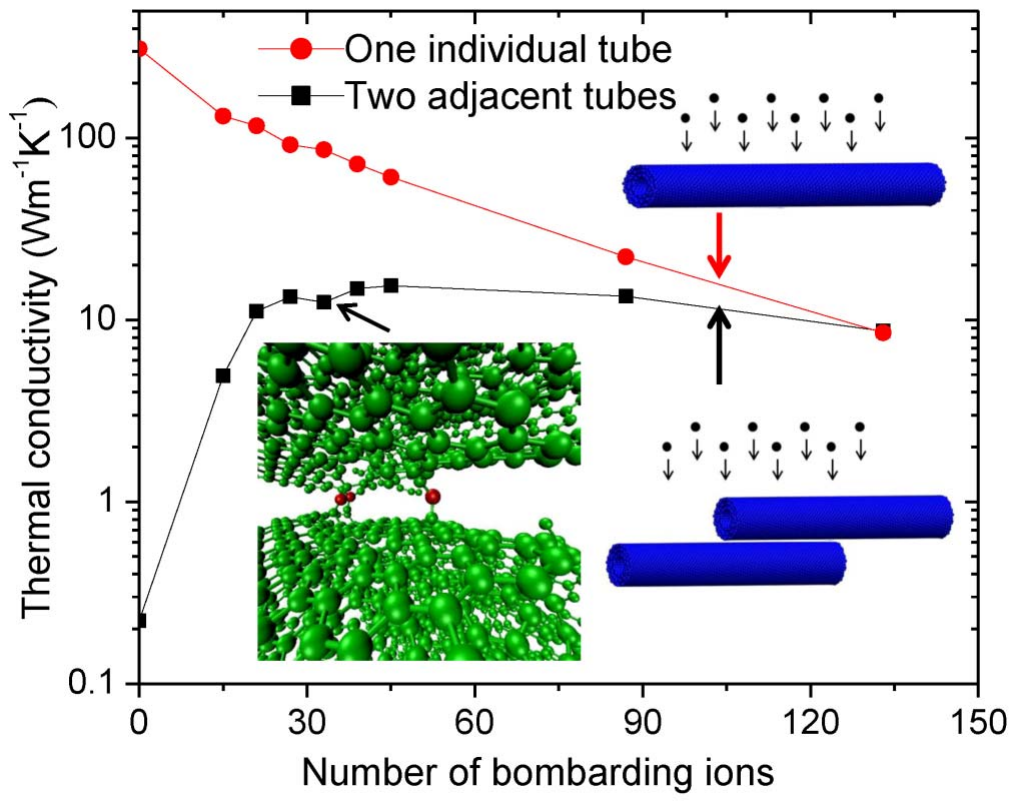
Calculated thermal conductivities of MWNTs. The comparison was made between an individual MWNT and two adjacent MWNTs as a function of increasing number of bombarding ions. MWNTs, each consisting of three walls, were simultaneously bombarded with ions and relaxed to allow formation of stable defects. The inset shows representative defects formed after bombardment with 33 ions. The red colored atoms refer to inter-tube displacements and green atoms denote lattice atoms.

Molecular dynamics (MD) simulations were used to understand what governs thermal property changes at the atomic scale^28^. To represent simplest networking of the tubes in a mat, two 20 nm long MWNTs were positioned 0.34 nm apart parallel to each other with 10 nm overlapping (see Fig. 4 for schematic). The separation distance between adjacent tubes was the same as the separation distance between individual walls in a MWNT, which was determined experimentally using high-resolution transmission electron microscopy. The modeling included two steps: C self-ion irradiation and thermal properties determination. Radiation damage was simulated using 500 eV C ion bombardment of adjacent nanotubes and then the Müller-Plathe method was used to calculate thermal conductivities^29^. A temperature gradient was built by exchanging velocity vectors of the atom with the highest kinetic energy at the “cold” end with the atom with the lowest kinetic energy at the “hot” end. This energy exchange is performed periodically every 0.005 ps. Thermal conductivities at 50 K are extracted from the equation: 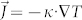, where 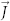 is the heat flux, κ is the thermal conductivity, and ∇T is the temperature gradient. Convergence of thermal conductivity with time means that a balance is reached between the energy transfer through velocity exchange and the temperature gradient-induced heat flux in the opposite direction (Supplementary Fig. 5).

It is not realistic to run MD simulations for high energy H ion irradiation since the required cell sizes are several orders of magnitude larger than computational limits. Furthermore, MD simulations do not consider electron excitation, which dominates stopping power of H ions at high energies. One method to alleviate this issue is to simulate radiation damage caused by ions of sub-keV energies, which reduces the required cell size but still can reveal the atomic scale details of damage cascade production. C self-ion bombardment, instead of H, was modeled because the majority of damage created in experiments comes from sub damage cascades formed during knocking of C atoms by the incident H atoms. Furthermore, at C incident energy of 500 eV, damage production is highly efficient due to energy dependence of nuclear stopping powers. Our modeling selected 3-wall MWNTs to include basic features of MWNTs. The selection of tube thickness, diameter and lengths will influence κ values of irradiated and unirradiated mats, but we did not follow true CNT structure used in experimental work because of computational resource limits. Our primary interest is to obtain general conclusion on irradiation effects by using simplified systems.

Figure 4 shows changes in calculated thermal conductivities with increasing number of bombarding ions. In an isolated CNT, κ decreases exponentially with increasing radiation damage, while in two adjacent CNTs κ first increases exponentially, saturates and then decreases at high damage levels. MD simulations confirm that inter-tube displacements efficiently promote tube-to-tube phonon transport between adjacent nanotubes, compensate for and exceed phonon scattering by defects, and consequently increase conductivity.

## Discussion

Through modeling we observed significant κ enhancement by a single inter-tube displacement created between two tubes. As it can be seen from Fig. 4, calculated thermal conductivity is enhanced by a factor of about 44 at 39 ions. Beyond that phonon-defect scattering-caused thermal conductivity loss within the tubes prevails over inter-tube displacement-assisted phonon transport.

Our modeling shows κ enhancement by two orders of magnitude, while the maximum enhancement from experimental measurement is about 5. The difference is due to the fact that MD simulations are based on very simplified structures with two perfectly aligned tubes. But in reality, tubes are randomly linked to each other. The tube lengths, wall thickness, tube-tube orientations, nature of random CNT networking, and incident ions/energies are very different between the modeling and the experiments. Furthermore, MD simulations are limited to very short time and length scales, and the scale difference between modeling and experiments still is a barrier for direct comparison. Structural relaxation and defect clustering occurring at longer time scale beyond MD simulation can be much more complicated. Therefore, the modeling in the present study is used to reveal fundamental mechanisms only.

**Figure 5:**
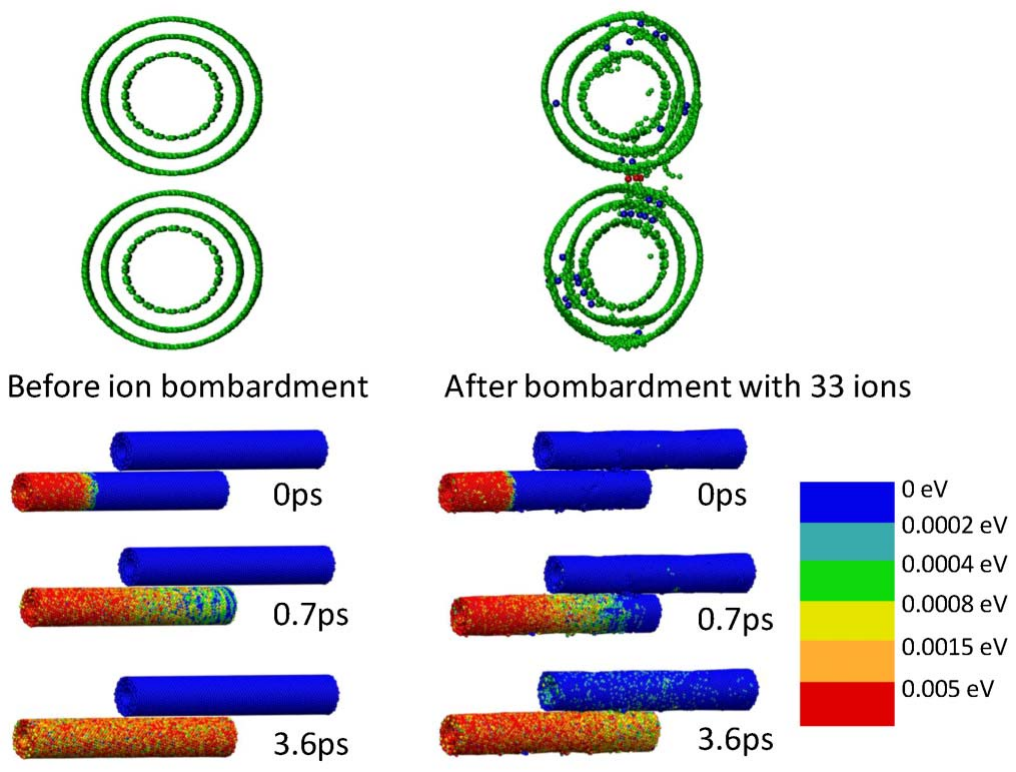
Distribution of kinetic energies in CNTs before and after irradiation with 500 eV C ions. The defects formed during ion bombardment are provided in blue (inter-wall displacements) and red (inter-tube displacements) in cross-sectional view of irradiated MWNTs. A 5 nm thick section of the nanotube on the left side of the bottom CNT is heated and time dependence of kinetic energy distribution is plotted.

Additional simulations were performed to visualize the difference in heat distribution in CNTs with or without radiation damage. Irradiation damage was introduced by bombarding two adjacent nanotubes with 33 C ions of 500 eV. Then a 5 nm thick section on the left side of the bottom nanotube was heated to 300 K, while the rest of the tube and the adjacent tube remained at 0.01 K. Figure 5 plots the distribution of kinetic energies after *t* = 0,0.7 and 3.6 *ps*. Irradiation induced inter-wall displacements (shown in blue) and inter-tube displacements (shown in red) are clearly visible. When heated unirradiated nanotube distributes the heat, it vibrates energetically without influencing the adjacent tube because of the inefficient phonon transport between two tubes. In irradiated nanotubes, distribution of heat is delayed by phonon-defect scattering, but inter-tube displacement-mediated phonon transport improves the heat transfer across two tubes and increases the temperature of the adjacent nanotube, which is not observed in unirradiated CNTs. The modeling results support the proposed competing mechanisms.

Because of high mobility of point defects at elevated temperatures, their removal during annealing requires an activation of 0.36 eV^21^. Displaced carbon atoms confined by adjacent tubes, on the other hand, are less mobile and their removal involves higher activation energy. Thus phonon transport across the tubes remains efficient and defect-phonon scattering within the tube is reduced. Both effects lead to further κ enhancement after post-irradiation annealing, as what observed from Fig. 3. Additionally, Fig. 3 compares thermal conductivities of unirradiated mats before and after annealing at 1173 K. High temperature annealing, when not combined with irradiation, causes almost negligible thermal conductivity changes. Observed large difference between irradiated and unirradiated CNT mats suggests that ion irradiation can manipulate thermal properties more than traditional high temperature annealing, and the combination of ion irradiation and heat treatment are complementary for thermal conductivity manipulation. This also excludes the unlikely possibility that, assuming the presence of defects in as-fabricated CNTs, thermal conductivity enhancement is caused by defect removal by either beam heating or dynamic defect interactions during ion irradiation.

## Methods

### Annealing

Annealing was performed in a quartz tube with a vacuum better than 1 × 10^−6^ torr. Prior to the annealing, the tube was purged with Ar gas multiple times. Mat-bearing quartz boats were pushed into the hot zone set to the desired temperature and quickly pulled upon completion of the annealing step.

### Thermal diffusivity measurements

Thermal diffusivities of specimens were measured using the laser flash method in NETZSCH LFA 447 NanoFlash tool. In this method the front surface of the mat is heated by the short intensive Xe pulse and the resulting temperature increase on the opposite side of the mat is measured using an infra-red (IR) InSb detector. CNT mats, with dimensions of 12.7 mm × 12.7 mm, are positioned into a carrier in sample holder plate, equipped with a temperature sensor. Furnace, integrated into the sample changer of the system, allows conducting temperature-dependent measurements. Thermal diffusivities were calculated using the following expression: 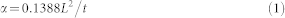
where *α* is the thermal diffusivity of the material, *L* the sample thickness, and *t* the time it takes for the opposite side of the specimen to reach half of the maximum temperature^30^. This technique has been previously used to characterize CNT films^31^. Temperature dependent specific heat of CNTs, both single- and multi-walled, converges with that of graphene and graphite at temperatures exceeding 100 K^32^, and the previously reported heat capacity data are used to convert thermal diffusivities (Supplementary Fig. 4) of CNTs to thermal conductivity values^33^.

### MD simulations

The MD simulations were performed by using LAMMPS (Large-scale Atomic Molecular Massively Parallel Simulator)^28^. The C-C covalent bonds were described by a 3-DTersoff potential combined with Ziegler-Biersack-Littmark (ZBL) universal screening potential at short interatomic distances^34^,^21^. In the Müller-Plathe method, thermal conductivity value is calculated using: 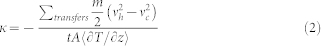
where *ν_h_* and *ν_c_* are velocity of C atoms at “hot” and “cold” sides, respectively, *m* is atomic mass of carbon, *t* is simulation time, *A* is the tube cross-section, *T* is temperature, and *z* is the distance between two ends. The advantages of this method include quick convergence with reduced fluctuation, precise knowledge of the imposed heat flux, and conservation of both kinetic energy and linear momentum.

Instantaneous local temperatures at “hot” and “cold” sides are calculated from: 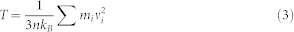
where the sum is taken over the section with a total *n* atoms, *m_i_* is the atomic mass of carbon, *ν_i_* is the velocity of C atoms, and *k_B_* is the Boltzmann's constant.

## Author Contributions

A.A. and L.S. designed/performed the experiments, analyzed data, and co-wrote the manuscript; D.C. and L.S. carried out molecular dynamics simulations. All authors discussed the results and commented on the manuscript.

## Supplementary Material

Supplementary Information Supplementary documents
