# Supplementary material for: Phonon transport assisted by inter-tube carbon displacements in carbon nanotube mats
Source: Sci Rep. 2013 Sep 27;3:2774. doi: 10.1038/srep02774 (PMC3784949; doi:10.1038/srep02774)
Supplement: Supplementary Information — Supplementary documents [file srep02774-s1.doc]

**Supplementary materials for**

**Phonon transport assisted by inter-tube carbon displacements in carbon nanotube mats**

A. Aitkaliyeva^1^, D. Chen^2^, L. Shao^1,2*^

^1^Materials Science and Engineering Program, Texas A&M University, College Station, Texas, 77843, USA

^2^Department of Nuclear Engineering, Texas A&M University, College Station, Texas, 77843, USA

^*^Corresponding author. Email: lshao@tamu.edu(L. Shao)

**Structural characterization**

The free-standing carbon nanotube (CNT) mats (Nano-Lab Inc., Waltham, MA) were fabricated by (1) suspending nanotube powder in de-ionized water with surfactants; (2) sonicating and filtering on a membrane; and (3) drying and removing continuous nanotube mats from the supporting membrane. Before and after ion irradiation, CNT mats were characterized in FEI Quanta 600 FE scanning electron microscope (SEM) and FEI TECNAI G^2^ F20 ST transmission electron microscope (TEM), operated at accelerating voltages of 30 kV and 200 kV, respectively. For TEM characterization, CNT mats were embedded in resin, sectioned using microtome, and deposited on formvar coated copper grids.

Figure 1 shows SEM micrographs of CNT mats before and after irradiation with H ions. Figures 1b-d show the micrographs of the mats irradiated with 1.5 MeV, 2 MeV, and 3 MeV H ions to their respective highest ion fluence values. Structural integrity of nanotubes was preserved at all irradiation conditions and no obvious densification of the mats was observed. Thickness of unirradiated and irradiated CNT mats was measured and compared to ensure that irradiation does not cause any fluctuations. The results indicate that thermal properties change is not the result of densification caused thickness changes.

**Figure 1. SEM micrographs of CNT mats.** **a**, before irradiation. **b,** after ion irradiation with 1.5 MeV H to a fluence of 3.5×10^15^/cm^2^. **c,** 2 MeV H to 4.5×10^15^/cm^2^. **d**, 3 MeV H to 6×10^15^/cm^2^. Scale bar denotes 1 μm.

**Figure 2. TEM micrographs of CNTs.** **a,** before irradiation. **b,** after ion irradiation with 1.5 MeV H to a fluence of 3.5×10^15^/cm^2^. **c,** 2 MeV H to 4.5×10^15^/cm^2^. **d,** 3 MeV H to 6×10^15^/cm^2^.

Figure 2 shows TEM micrographs of CNTs prior to and after ion irradiation and their corresponding filtered images after Fourier transformation in insets. TE micrographs show well-ordered structures and good crystalline quality of nanotubes. Formation of amorphous zones, significant distortion, and breaking of basal planes are not detected. This suggests that thermal properties are not modified by structural amorphization, and that C displacements and point defects are primarily responsible for the observed changes.

**Thermal diffusivity measurements**

Temperature dependence of experimentally determined thermal diffusivities of CNT mats before and after irradiation is shown in Fig. 3. Before irradiation diffusivity value was at about 1×10^-7^ m^2^/s between 300 K and 450 K. As shown in Fig. 3a, after irradiation with 1.5 MeV H ions, diffusivities of irradiated mats are enhanced, with the maximum enhancement by a factor of 5.3 attained at 1.4×10^15^/cm^2^. However, after irradiation to 3.5×10^15^/cm^2^, the diffusivities trend starts to decrease, which shows the existence of a fluence range where α values can be maximally increased.

In a laser flash method^1,2^, thermal diffusivity of the specimen can be determined without the knowledge of the amount of energy deposited on the surface. However, it is required to calculate both specific heat and thermal conductivity of the material. Specific heats of single- and multi-walled nanotubes, graphene, and graphite have different temperature dependence at low temperatures (< 80 K) but at temperatures exceeding 100 K specific heat curves of all materials converge^3^. Temperature dependent specific heat of graphite, based on previously reported data^4^, is provided in Fig. 4. As it can be seen, measured specific heat continuously increases with temperature, with a dramatic increase observed at low temperatures. Thermal diffusivities of the

**Figure 3. Temperature dependence of extracted thermal diffusivities of CNT mats.** **a,** before and after ion irradiation to various fluences with 1.5 MeV H. **b,** 2 MeV H. **c,** 3 MeV H ions.

**Figure 4. Specific heat of graphite as a function of temperature [4].**

mats were measured between 300 K and 450 K, thus specific heat of graphite can be used to convert thermal diffusivities of CNTs to thermal conductivity values.

**Modeling**

Comparison was performed between an individual multi-walled nanotube (MWNT) and two adjacent MWNTs. Each nanotube consisted of three walls, with a wall separation distance of 0.34 nm, and had an inner diameter of 1.63 nm. Two adjacent nanotubes were placed 0.34 nm apart and the temperature of the tubes was set to 100 K. Prior to 500 eV C ion bombardment tubes were allowed to relax at 100 K for 100 ps. Then dynamic defect interaction and recombination were continuously modeled for up to 2.5 ps, which is sufficiently long to cause saturation of defects in a relaxed structure. Positions of atoms, including defects, upon completion of this modeling step, were input into the subsequent thermal conductivity modeling.

A method developed by Müller-Plathe was used to model thermal conductivities of CNTs^5^. In this method the tube is divided into ten 5 nm thick sections and the first section on left side of the nanotube is defined as the “hot” side. The velocity vector of an atom with lowest energy at the “hot” side is then exchanged with that of an atom with highest energy at the “cold” side and the exchange is repeated every 0.005 ps. This produces an energy flux from “cold” towards “hot” sections of the nanotube and a temperature gradient in the opposite direction. Once a steady state is reached, the heat flux from the temperature gradient is balanced by the energy flux. This method ensures conservation of energy/momentum, and convergence of extracted thermal diffusivity.

Figure 5 shows the calculated thermal conductivities of unirradiated nanotubes and CNTs irradiated with 33 and 133 ions as a function of simulation time. All three curves decrease with increasing modeling time without any significant fluctuation. Thermal conductivity of irradiated CNTs saturate after 40 ps, while saturation in unirradiated CNTs is delayed. In the present study, conductivity values were selected at 70 ps, which is sufficiently long to obtain converged values.

**Figure 5. Calculated thermal conductivities of CNTs as a function of simulation time.**

**References:**

[1] Parker, J. W., Jenkins, R. J., Butler, C. P., Abbott, G. L., Flash method of determining thermal diffusivity, heat capacity, and thermal conductivity, *J. Appl. Phys.* **32,** 1679-1684 (1961).

[2] NETZSCH, Thermal diffusivity-Thermal conductivity: Methods, Technique, Applications.

[3] Hone, J., Batlogg, B. Benes, Z., Johnson, A. T., Fischer, J. E., Quantized phonon spectrum of single-wall carbon nanotubes, *Science* **289,** 1730-1733 (2000).

[4] Nihira, T., Iwata, T., Temperature dependence of lattice vibrations and analysis of the specific heat of graphite, *Phys. Rev. B* **68,** 134305(1)-(16) (2003).

[5] Müller-Plathe, F., A simple nonequilibrium molecular dynamics method for calculating the thermal conductivity, *J. Chem. Phys.* **106,** 6082-6085 (1997).
